# Shift in dominant genotypes of Japanese encephalitis virus and its impact on current vaccination strategies

**DOI:** 10.3389/fmicb.2023.1302101

**Published:** 2023-11-17

**Authors:** Qiqi Xia, Yang Yang, Yan Zhang, Lujia Zhou, Xiaochun Ma, Changguang Xiao, Junjie Zhang, Zongjie Li, Ke Liu, Beibei Li, Donghua Shao, Yafeng Qiu, Jianchao Wei, Zhiyong Ma

**Affiliations:** ^1^Shanghai Veterinary Research Institute, Chinese Academy of Agricultural Sciences, Shanghai, China; ^2^College of Veterinary Medicine, Nanjing Agricultural University, Nanjing, China

**Keywords:** Japanese encephalitis virus, genotype shift, vaccine, cross-protection, virulence

## Abstract

Japanese encephalitis (JE) is a zoonotic ailment from the Japanese encephalitis virus (JEV). JEV belongs to the flavivirus genus and is categorized into a solitary serotype consisting of five genetically diverse genotypes (I, II, III, IV, and V). The JEV genotype III (GIII) was the prevailing strain responsible for multiple outbreaks in countries endemic to JEV until 1990. In recent years, significant improvements have occurred in the epidemiology of JE, encompassing the geographical expansion of the epidemic zone and the displacement of prevailing genotypes. The dominant genotype of the JEV has undergone a progressive shift from GIII to GI due to variations in its adaptability within avian populations. From 2021 to 2022, Australia encountered an epidemic of viral encephalitis resulting from infection with the GIV JEV pathogen. The current human viral encephalitis caused by GIV JEV is the initial outbreak since its initial discovery in Indonesia during the late 1970s. Furthermore, following a time frame of 50 years, the detection and isolation of GV JEV have been reported in *Culex* mosquitoes across China and South Korea. Evidence suggests that the prevalence of GIV and GV JEV epidemic regions may be on the rise, posing a significant threat to public safety and the sustainable growth of animal husbandry. The global approach to preventing and managing JE predominantly revolves around utilizing the GIII strain vaccine for vaccination purposes. Nevertheless, research has demonstrated that the antibodies generated by the GIII strain vaccine exhibit limited capacity to neutralize the GI and GV strains. Consequently, these antibodies cannot protect against JEV challenge caused by animal GI and GV strains. The limited cross-protective and neutralizing effects observed between various genotypes may be attributed to the low homology of the E protein with other genotypes. In addition, due to the GIV JEV outbreak in Australia, further experiments are needed to evaluate the protective efficiency of the current GIII based JE vaccine against GIV JEV. The alteration of the prevailing genotype of JEV and the subsequent enlargement of the geographical extent of the epidemic have presented novel obstacles in JE prevention and control. This paper examines the emerging features of the JE epidemic in recent years and the associated problems concerning prevention and control.

## 1 Introduction

Japanese encephalitis (JE) is a zoonotic illness spread by mosquitoes initially identified in Japan in 1871 in humans and animals. The Nakayama strain of the Japanese encephalitis virus (JEV), which causes JE, was first identified in a 1935 fatal human case (Solomon et al., 2000; Erlanger et al., 2009; Kulkarni et al., 2018). According to the World Health Organization, JE is prevalent mainly in large parts of the Pacific and Asia, with high incidence and risk, seriously threatening the health of humans and animals. It has been shown that JEV infection has affected over 24 countries and areas (Campbell et al., 2011; Mulvey et al., 2021), and more than 3 billion people are at risk of infection. During the initial months of 2022, JEV was determined to be the etiological agent responsible for the occurrence of stillborn and mummified piglets inside pig farming establishments located in the southeastern region of Australia. Later, instances of JEV infection in humans were detected across a geographically extensive area, spanning 4 states (van den Hurk et al., 2022).

JEV is a mosquito-borne zoonotic infection affecting vertebrates (pigs and birds) and invertebrates (mosquitoes). The two primary cycles of JEV transmission are the pig-associated rural domestic transmission cycle and the bird-associated wild transmission cycle (Oliveira et al., 2018). Several species of mosquitoes, such as *Armigeres* (Ar.), *Anopheles* (An.), *Aedes* (Ae.), and *Culex* (Cx.) mosquitoes can transmit JEV across different vertebrate hosts (de Wispelaere et al., 2015; Huang et al., 2016), whereas *Cx. tritaeniorhynchus* plays a significant role in JEV transmission (Hu and Grayston, 1962; Bendell, 1970; Vaughn and Hoke, 1992). Furthermore, it has been observed that JEV can be spread among pigs even in the absence of arthropod vectors. Pigs and avian species have a crucial role in the infection’s preservation, augmentation, and dissemination, whereas humans and equines serve as terminal hosts (Simpson et al., 1976; Nain et al., 2016).

More than 18 species of vertebrates, including humans, pigs, birds, water buffalos, raccoons, monkeys, wild boars, goats, sheep, frogs, snakes, bats, cats, cattle, ducks, chickens, dogs, and horses, are susceptible to JEV infection and demonstrate viremia and/or seroconversion after natural or experimental infection. However, most ailments are subclinical, except in humans, pigs, and horses (Kumar et al., 2018). Most human infections caused by JEV manifest without noticeable symptoms, while children and older individuals may experience mild conditions that can potentially escalate to severe encephalitis. The disease exhibits an incubation period ranging from 4–15 days. Individuals affected by JE may initially present with nonspecific symptoms, including myalgia, vomiting, diarrhea, nausea, headache, and fever (Solomon, 2004; Sips et al., 2012). These symptoms may be accompanied by acute encephalitis, fever, neck rigidity, and convulsions. Speech impairment can manifest in patients, accompanied by subsequent neurological symptoms, including seizures, meningitis, and Parkinson’s syndrome. The fatality rate of JE is estimated to be around 20–30%, but a substantial proportion of survivors, ranging from 30 to 50%, experience notable neurological complications (Campbell et al., 2011; Mulvey et al., 2021).

Swine are highly vulnerable to infection by JEV and exhibit characteristic clinical manifestations, including neurological symptoms associated with encephalitis in piglets, instances of abortion, stillbirth, and dystocia in pregnant sows, as well as orchitis in boars (Scherer et al., 1959c). In addition, the pathological changes in the dead pigs mainly occur in the brain; the meningoencephalitis parenchyma becomes hyperemic; the liver, spleen, kidney, and other organs become necrotic to varying degrees; and the testicles of the pigs become swollen and hyperemic (Zhang et al., 2015). Notably, in large-scale breeding plants, JEV infection significantly affects the fertility of pigs, and the risk of stillbirth and congenital malformation dramatically increases. This situation occurs periodically during the mosquito season (Takashima et al., 1988). However, it is only limited to countries with seasonal epidemics. In tropical and subtropical regions, mosquito vectors survive all year round. Sows produce positive serum before their first pregnancy to resist virus infection and reduce the outbreak of JEV in pigs (Lindahl et al., 2012).

JE currently lacks a specific pharmacological intervention, necessitating maintenance therapy as the primary approach (Sampath and Padmanabhan, 2009), including seizure control and ventilator support for those with respiratory failure by reducing and monitoring cerebral edema. By contrast, prevention is the main focus in farming, and treatment of JE is not advocated. Hence, immunization emerges as the optimal and viable approach for averting JE in human and animal populations. China presently employs two JEV vaccines, namely the SA14-14-2 live attenuated vaccine (LAV) and the inactivated P3 strain vaccine (IPV) (Hegde and Gore, 2017; Hills et al., 2019). Although the existing JE vaccine can prevent the current JEV and show specific immune effects, it is developed based on GIII JEV (Fan et al., 2012; Teng et al., 2013). However, the emerging GI and GV challenge the GIII-based vaccines.

## 2 Transmission of JEV

**Figure 1:**
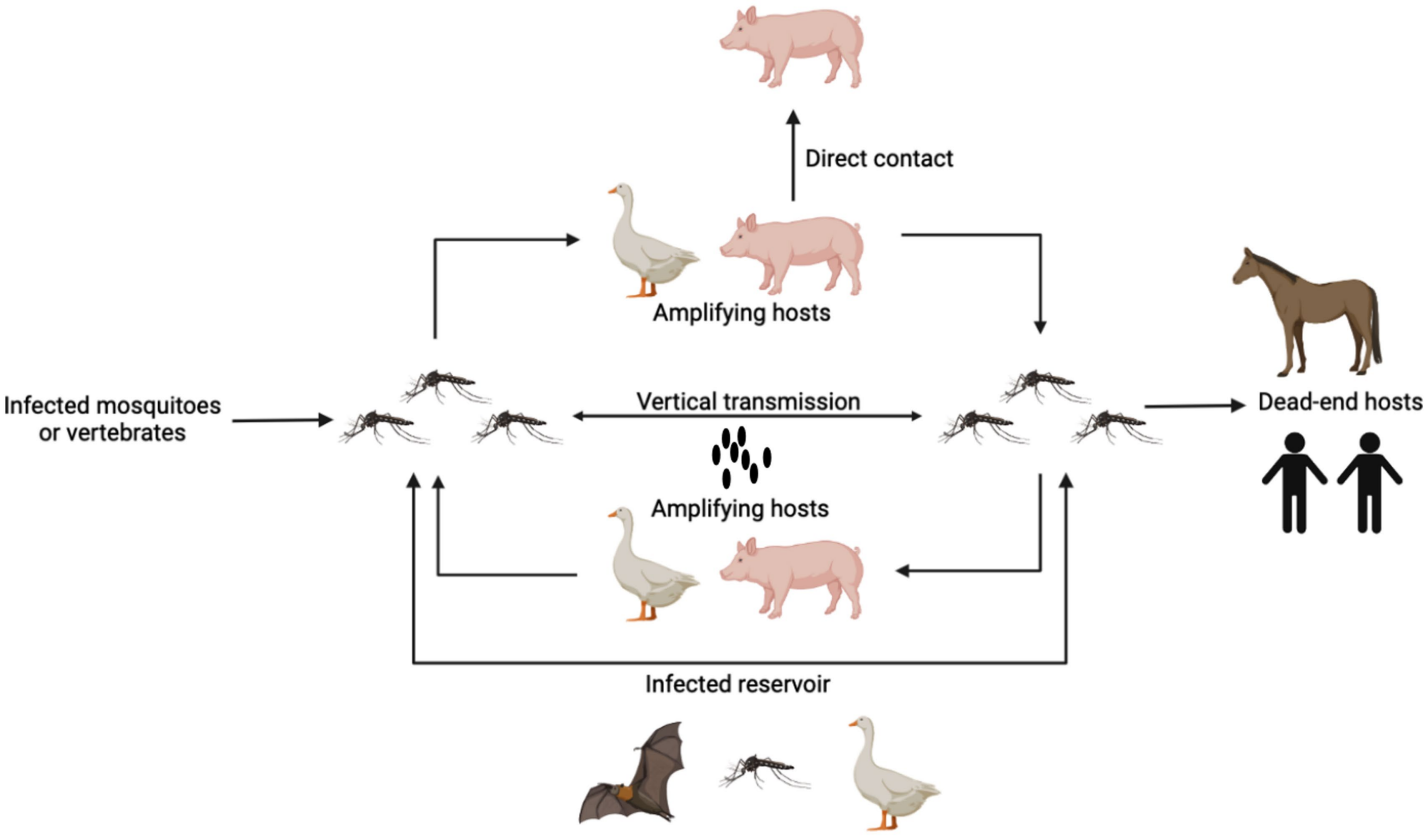
The pattern of JEV transmission. Mosquitoes carry the JEV after sucking blood and transmit the virus between animals via biting. Mosquitoes can also carry the virus through vertical transmission via the desiccation-resistant eggs. Pigs and birds are reservoir and amplification hosts, which produce high-level viremia for a long time. Pigs also can be infected with JEV through direct contact. Human and horses are the dead-end hosts. Other animals such as bats can also be infected and carry JEVs for a long time.

As a zoonotic infection spread by mosquitoes, JEV depends on arthropods, primarily mosquitoes, to finish its transmission cycle, which is divided into two parts: the pig-associated rural domestic transmission cycle and the bird-associated wild transmission cycle (Oliveira et al., 2018). The coexistence or separate occurrence of these two transmission cycles is contingent upon the biological characteristics of vectors, hosts, and environmental variables (Johansson et al., 2013). Mosquitoes, pigs, and birds are involved in JEV transmission and have different roles (Figure 1). Mosquitoes, such as *Cx. tritaeniorhynchus* carry the JEV after sucking blood and transmit the virus between animals via biting, playing the role of a transmission vector (van den Hurk et al., 2009). Mosquitoes can also carry the virus through vertical transmission via the desiccation-resistant eggs (Rosen et al., 1978; Takashima and Rosen, 1989). Avian and porcine species exhibit prolonged viremia following infection with JEV, serving as reservoir and amplification hosts. Furthermore, JEV can endure in the tonsils of JEV-infected pigs for at least 25 days. Additionally, it can be transmitted among animals without the involvement of any vectors.

According to the impact of climate and season on the disease, the prevalence of JEV can be divided into two modes: year-round persistent epidemic and seasonal epidemic (Umenai et al., 1985). Persistent epidemics throughout the year are mostly manifested in tropical regions such as Brunei, Cambodia, Indonesia, Malaysia, Papua New Guinea, the Philippines, Singapore, and Sri Lanka; However, seasonal epidemics are mainly prevalent in China and some northern regions, such as Bangladesh, Bhutan, South Korea, Nepal, Japan, Pakistan, North Korea, Russia, South Korea and Vietnam, Thailand, and northern India (Umenai et al., 1985; Wang and Liang, 2015). JEV is an arbovirus, with Culex mosquitoes as its main vector insects (Pandit et al., 2018), which can persist throughout the year and become seasonal depending on climate and geographical location. In addition, infected mosquitoes can also spread in temperate regions and help the virus overwinter through vertical transmission (Pandit et al., 2018; Pearce et al., 2018). Summer is rainy, making it easy for mosquito vectors to breed, so there may be a major outbreak of JE. Although the peak of JE incidence occurs only after the start of the rainy season in sporadic tropical regions, Solomon et al. found that the occurrence of JE is more closely related to temperature through statistical analysis of JE cases in the southern and northern regions with consistent precipitation (Vaughn and Hoke, 1992; Solomon et al., 2000). JE often occurs in summer, but it is not limited to the season when mosquitoes are active. The biosecurity threat brought about by climate change requires people to face disease prevention and control with a brand new attitude. Conventional prevention and control ideas are no longer able to cope with the current diversified transmission routes of diseases, and the geographical expansion of mosquitoes caused by global warming has posed an increasingly serious global threat to humanity.

### 2.1 Mosquitoes

Mosquitoes are biologically significant insects that transmit flavivirus, including JEV, Yellow fever virus, Dengue virus, West Nile virus, and Zika virus, other vector-borne viruses (Andriessen et al., 2015; Fortuna et al., 2015; Paradkar et al., 2015). During the typical progression of viral infection, the virus is acquired through ingestion during a blood meal. Subsequently, it faces various obstacles in the midgut, such as infection and escape barriers, which it must successfully overcome (Bosio et al., 2000; Salazar et al., 2007). Viral multiplication ensues upon entry into the midgut epithelial cells, leading to subsequent dissemination of the virus to the hemocoel (Franz et al., 2015; Lee et al., 2019). The hemocoel is an open bodily cavity serving as a conduit for hemolymph circulation. Consequently, following the introduction of the virus into the hemolymph, it can disseminate to many secondary tissues, such as neural tissue, muscles, trachea, adipose body, and salivary gland, through the circulation of the hemolymph (Salazar et al., 2007; Xi et al., 2008). For the mosquito to be capable of transmitting the virus to other vertebrate hosts, it must first infect the salivary glands (Chauhan et al., 2012).

A diverse array of mosquito species has the potential to serve as vectors for the transmission of JEV. There are 14 mosquitoes belonging to five different genera and species were found to have the capability to transmit JEV. They are *Cx. vishnui*, *Cx. Tritaeniorhyncus*, *Cx. sitiens*, *Cx. quinquefasciatus*, *Cx. pseudovishnui*, *Cx. pipiens*, *Cx. gelidus*, *Cx. fuscocephala*, *Cx. bitaeniorhynchus*, *Cx. annulirostris*, *Armigeres subalbatus* (Ar.), *Ae. vigilax*, *Ae. vexans*, and *Ae. albopictus* (Auerswald et al., 2021). *Cx*. *tritaeniorhynchus* is the primary vector for JEV transmission (Sudeep, 2014; Oliveira et al., 2018; Pearce et al., 2018). Furthermore, it should be noted that many additional species, including *Cx. quinquefasciatus, Cx. annulirostris, Cx. gelidus, Cx. fuscocephala,* and *Cx. bitaeniorhynchus* can potentially be significant contributors to the transmission of JEV in specific geographical areas (Ching et al., 1970; Auerswald et al., 2021).

Mosquitoes are distributed worldwide. The *Cx. tritaeniorhynchus*, the most crucial carrier of JEV, is present in Southeast Asia, the Middle East, Africa, and Europe (Longbottom et al., 2017). In recent times, the presence of *Cx. tritaeniorhynchus*, a mosquito species known to transmit JEV, has been observed in the northern region of Australia. This area is characterized by a significant population of wading birds and feral pigs, contributing to an elevated risk of JEV establishment in this geographical location (Lessard et al., 2021).

The mosquito species *Cx. annulirostris* is widely recognized as the principal vector of JEV and is believed to inhabit nearly 80% of the landmass in Australia (van den Hurk et al., 2022). The species *Cx. annulirostris* has been linked to past JEV occurrences in the Torres Strait region of Australia, as documented by (Hanna et al., 1999). These outbreaks have been observed in various locations, some areas in Southeast Asia and across northern Australia (Ritchie et al., 1997). In the past few years, Australia has experienced a significant outbreak of JEV, with indications suggesting a potential association with mosquito transmission. There is a possibility that other Culex species may play a role in the transmission cycles of JEV in Australia. A further strain of the JEV GIV, which bears a high similarity of 98% in nucleotide identity to the Tiwi Islands virus, was identified in *Cx. gelidus* mosquitoes captured in Morobe Province, Papua New Guinea, in 2019 (D.T. Williams reported these findings in an unpublished study). The range of this species in northern Australia has been demonstrated to be widespread (Williams et al., 2005).

The *Cx*. *vishnui* was isolated from mosquitoes in India (Gajanana et al., 1997), Thailand (Leake et al., 1986), and Indonesia (Olson et al., 1985) and is mainly distributed in some countries in Southeast Asia (Vythilingam et al., 1995). *Cx. bitaeniorhynchus* was found in mosquitoes in India, South Korea (Kim H. C. et al., 2015), and Malaysia (Vythilingam et al., 1995). In addition, several JEVs were isolated from some field mosquitoes in Malaysia. *Cx. fusocephala* and *Cx. gelidus* were isolated from mosquito samples in Southeast Asia (Gould et al., 1974; Mourya et al., 1989; Arunachalam et al., 2009). The species under consideration primarily inhabit Southeast Asia and have a distribution that encompasses Japan and China in the northeast, Timor in the south, and Pakistan in the west. However, *Cx. gelidus* is also found in the northern Australian region (van den Hurk et al., 2003; Ritchie et al., 2007). When JEV broke out in South Korea (Seo et al., 2013; Kim H. C. et al., 2015), Italy (Ravanini et al., 2012), and in some places in China (Kim H. et al., 2015), JEV was detected in *Cx. pipiens*. *Cx. pipiens* were also found in some countries in East Asia, such as China (Huang, 1982), South Korea, and Japan (Buescher et al., 1959). The *Cx. sitiens* was isolated from mosquitoes in Malaysia (Vythilingam et al., 1995), Papua New Guinea (Johansen et al., 2000), Taiwan (Weng et al., 2005), and Australia (Johansen et al., 2001; Van Den Hurk et al., 2006). The distribution of the item encompassed East and South Asia, as well as northern Australasia (Auerswald et al., 2021). Furthermore, it has been determined that *Culex sitiens* is a proficient carrier of JEV within the Australian context (van den Hurk et al., 2003). *Cx. tritaeniorhynchus* is widely recognized as the principal vector for JEV in numerous countries. JEV has been isolated from several nations and locations, including Japan (Sabin, 1947), China (Feng et al., 2012; Shi et al., 2019), Indonesia (Van Peenen et al., 1975; Olson et al., 1985), India (Mourya et al., 1989; Gajanana et al., 1997), Malaysia (Simpson et al., 1970; Vythilingam et al., 1997), Thailand (Simasathien et al., 1972), South Korea (Pant et al., 1994), Vietnam (Thi-Kim-Thoa et al., 1974), Cambodia (Duong et al., 2017), and Singapore (Yap et al., 2020). Finally, *Cx. quinquefasciatus* differs from the others in geographic distribution; it is mainly distributed in Madagascar, North America, and Argentina (Huang et al., 2015, 2016). JEV was successfully isolated from mosquitoes in India (Mourya et al., 1989), Vietnam (Thi-Kim-Thoa et al., 1974), Taiwan (Weng et al., 1999), and Thailand (Nitatpattana et al., 2005). The widespread distribution of these mosquitoes worldwide is closely related to the endemic areas of JEV.

### 2.2 Birds

Avian species are prone to JEV infection. The majority of infections have subclinical manifestations; however, they have the potential to induce growth suppression and mortality in specific organisms (Nemeth et al., 2012; Xiao et al., 2018b). Since 1958, the function of birds as reservoir hosts for JEV has been acknowledged (Gresser et al., 1958; Scherer et al., 1959a). Birds have also been identified as essential transmission hosts for JEV (Hameed et al., 2021) and are crucial in the bird-related wild transmission cycle. In general, it has been shown that over 90 species of both domesticated and wild avian creatures exhibit viremia and/or seroconversion after either natural or artificial infection (van den Hurk et al., 2009; Nemeth et al., 2012). Within this species group, it is widely acknowledged that wild ardeid waterbirds, specifically egrets and herons, play a significant role as hosts for transmitting JEV (Soman et al., 1977). These waterbirds exhibit a high susceptibility to JEV infection, producing substantial levels of viremia that can persist for up to four days. Consequently, they serve as a crucial source of infection for mosquitoes (Gresser et al., 1958).

A notable correlation between the degree of JEV viremia and the age of ducklings and chickens was observed while examining the viremia spectrum (Cleton et al., 2014). The viremia observed in juvenile avian specimens exhibits a substantial degree of elevation, akin to the viremia levels documented in previous reports (Dhanda et al., 1977; Soman et al., 1977). Previous studies showed that the viremia (≥10^4^ PFU/mL) in 1–3-day-old chicks led to 100% transmission of *Cx. tricornicorhynchus* mosquitoes and approximately 10% of mosquito infections were caused by birds with edible viremia of only approximately 10^2^ PFU/mL (Dhanda et al., 1977). Xiao et al. (2018a) have recently proposed variations in the reactions and susceptibilities of ducklings to the JEV infection, which can be attributed to different strains of JEV. A laboratory tested the pathogenicities of seven GI and GIII JEV strains in newly hatched domestic ducklings; the highest proportion (69.2%) of viremic individuals and the highest viremia titer (10^3.4 ± 1.3^) were obtained in ducklings inoculated with the SD12 strain. The findings indicated variations in the sensitivity and vulnerability of ducklings to JEV infection across different strains of JEV (Xiao et al., 2018b). In a particular investigation, the ducklings underwent inoculation through subcutaneous injection (Xiao et al., 2018b), whereas Di et al. (2020) inoculated the ducklings through JEV-infected mosquito bites. The data above prove that infection with JEV by mosquito bites leads to mortality linked to viral encephalitis in recently hatched domestic ducklings. This demonstrates the potential of JEV to cause disease in domestic ducklings in their natural environment. The viremia observed in juvenile birds is sufficiently elevated to result in a transmission rate of 50 to 100% for *Cx. pipiens* mosquitoes (Dhanda et al., 1977; Turell et al., 2006) are the known effective vector of JEV (Hameed et al., 2019). In addition, the migratory birds that fly seasonally between tropical, subtropical, and temperate regions and across oceans can also spread JEV to a new geographic area (Samy et al., 2016; Filgueira and Lannes, 2019).

### 2.3 Pigs

Pigs, encompassing both domesticated pigs and wild boars, exhibit susceptibility to infection caused by JEV. In regions where the disease is prevalent, the seroprevalence of JEV typically exhibits a high occurrence rate ranging from 98 to 100% following the conclusion of the mosquito season. Infection with JEV typically does not manifest any discernible clinical symptoms in juvenile and mature swine. However, it is worth noting that breeding pigs might still experience reproductive diseases, including orchitis, stillbirths, and mummified fetuses. Additionally, piglets may also suffer from encephalitis. Particularly, pigs can develop hyperviremia within a few days after JEV infection, and play a role in the pig-associated rural domestic transmission cycle. The viremia levels in the JEV-infected pigs rise to a point where they can infect mosquitoes for up to four days (Hurlbut and Thomas, 1950). Consequently, pigs are involved in the transmission of pig-mosquito-pig or pig-mosquito-human (Scherer et al., 1959b; Park et al., 2018).

In regions where JEV is prevalent, the seroprevalence of JEV is typically observed to be high, ranging from 98 to 100%, which suggests that most pigs have sufficient virus for infecting biting mosquitoes (Hurlbut and Thomas, 1950). When pigs are infected with JEV, the virus can be detected in their tonsils for at least 25 days. Furthermore, it is worth noting that pigs can transmit diseases to one another without the involvement of vectors, such as mosquitoes or ticks. This transmission can occur through direct touch, as pigs are particularly vulnerable to infections that can be transmitted by the mouth or nose.

Pigs are utilized for JEV maintenance and multiplication, as well as for facilitating transmission through the pig-mosquito-pig or pig-mosquito-human routes (Scherer et al., 1959b; Park et al., 2018). Hence, it is imperative to administer vaccinations to pig herds to mitigate the likelihood of JEV infection among pigs and consequent outbreaks in the human population (Mulvey et al., 2021). At the same time, with the rise in modern and large-scale pig farming, traditional breeding methods are gradually being phased out, and the continuous updating of breeding equipment and environment has reduced the contact opportunities between hosts and transmission media. The management of pigs’ involvement in the transmission cycle of JEV is gradually being regulated.

### 2.4 Other hosts

Besides swine infection, JEV has the potential to induce disease in various domesticated species such as goats, cattle, buffalo, and horses, as well as in wild animals like marsupials. These animals are regarded as dead-end hosts in the context of JEV transmission (Mulvey et al., 2021). Most JEV infections in horses appear to be subclinical, but morbidity persists. Reports from Japan in the 1940s indicated that JEV epidemics occurred frequently, affecting thousands of horses (Sugiura and Shimada, 1999). Reports of JEV infection in horses are rare because of inactivated vaccines (Goto, 1976) and reduced horse populations. Fever and neurological symptoms, loss of appetite, and ataxia have been reported in infected horses, with one horse dying within 3 days of onset and another being euthanized after receiving treatment (Mansfield et al., 2017). Cattle have similar clinical manifestations to horses after infection with JEV, with early symptoms of depression and loss of appetite. Signs of neurological dysfunction, such as hovering in place or inability to walk, develop over the next few days. In addition, the reproductive capacity of cattle is also damaged (Katayama et al., 2013; Kako et al., 2014). Additional species of domesticated animals, including goats, dogs, and cats, exhibit subclinical infections characterized by seropositivity that becomes evident solely subsequent to infection, while remaining asymptomatic (Mall et al., 1995). In addition, JEV was also isolated from brain samples of seals suffering from lethal encephalitis in a Chinese aquarium, and JEV might disseminate through infected zoo animals (Li et al., 2019).

JEV antibodies have also been reported in reptiles, such as snakes, frogs, and lizards (Kawasaki et al., 1970; Shortridge et al., 1974; Li et al., 2019). One of these studies found that JEV antibodies were detected in snakes, with the antibody levels in serum samples collected in spring and summer significantly higher than those contained in autumn and winter. It is suggested that snakes may be an essential host for JEV overwintering (Shortridge et al., 1974). Bats, which are crucial hosts for a variety of zoonotic viruses, can also be infected by JEV, and some species of bats can carry the virus for a long time (George et al., 2011); the virus can be transmitted vertically to the next generation through the placenta (Sulkin et al., 1964; Liu et al., 2013), have identified multiple strains of JEV that have been obtained from bats. However, the precise involvement of bats in the life cycle of JEV remains uncertain and necessitates additional investigation. There are many hosts of JEV, and it exists widely in nature. JEV can infect many wild animals, but there are few related studies, and the infection is difficult to observe and record. Therefore, the harm caused to animal health requires us to conduct more in-depth investigations.

## 3 Regions and evolution of JEV

**Figure 2:**
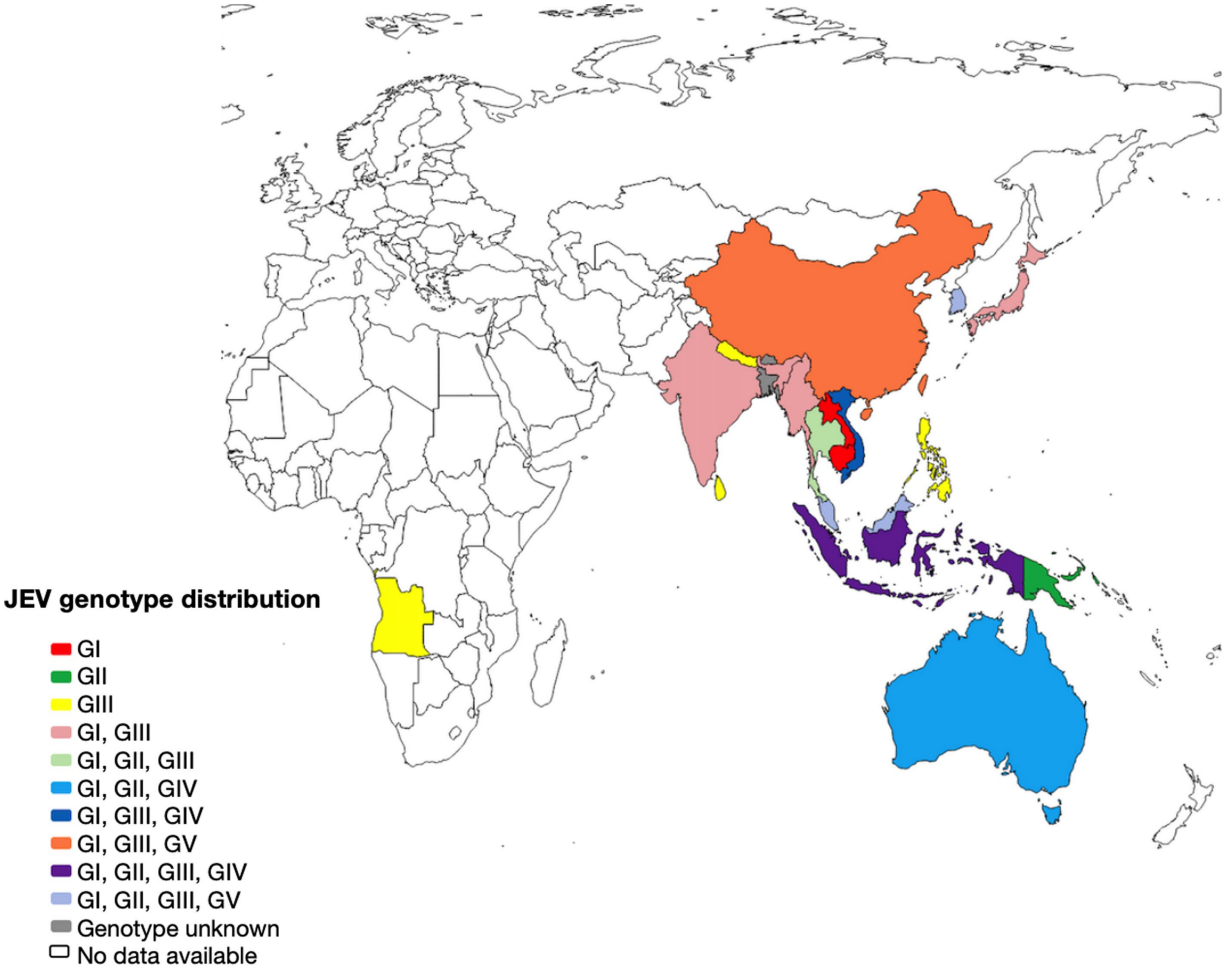
The geographical distribution of Japanese encephalitis virus. Map of the distribution of JEV genotypes based on available 5’UTR, structural and non structural protein sequences.

The first cases of epidemic encephalitis were detected in Japan beginning in 1871, and the first strain of JEV (Nakayama strain) was isolated in 1935 (Lewis et al., 1947). Since then, the virus has been found throughout much of Asia, with the geographic boundaries of viral activity extending westward to Pakistan, northward to Siberia, eastward to Saipan, and southward to Australia (Baig, 2013). JEV has only one serotype, but it can be classified into five genotypes according to the full-length genome sequence or E gene sequence, namely genotypes I, II, III, IV, and V (GI, GII, GIII, GIV, and GV) (Figure 2). From 1935 until the 1990s, type GIII was the most widespread genotype in most of Asia, particularly China, and has historically been the source of many common JE strains (Zheng et al., 2012). The earliest isolate of type GI was found in Cambodia in 1967, and the first strain of type GI JEV in China was discovered in 1979 (Wang et al., 2007). GII JEV is prevalent mainly in Australia, Malaysia, and Indonesia (Schuh et al., 2010). GIV JEV has been isolated in Indonesia, Australia, and Vietnam and caused a widespread outbreak in Australia in 2022 (Mackenzie et al., 2022). The GV JEV Muar strain was initially obtained from patients in Malaya in 1952 and has since been isolated and characterized in Korea and China (Li et al., 2011; Takhampunya et al., 2011).

**Figure 3:**
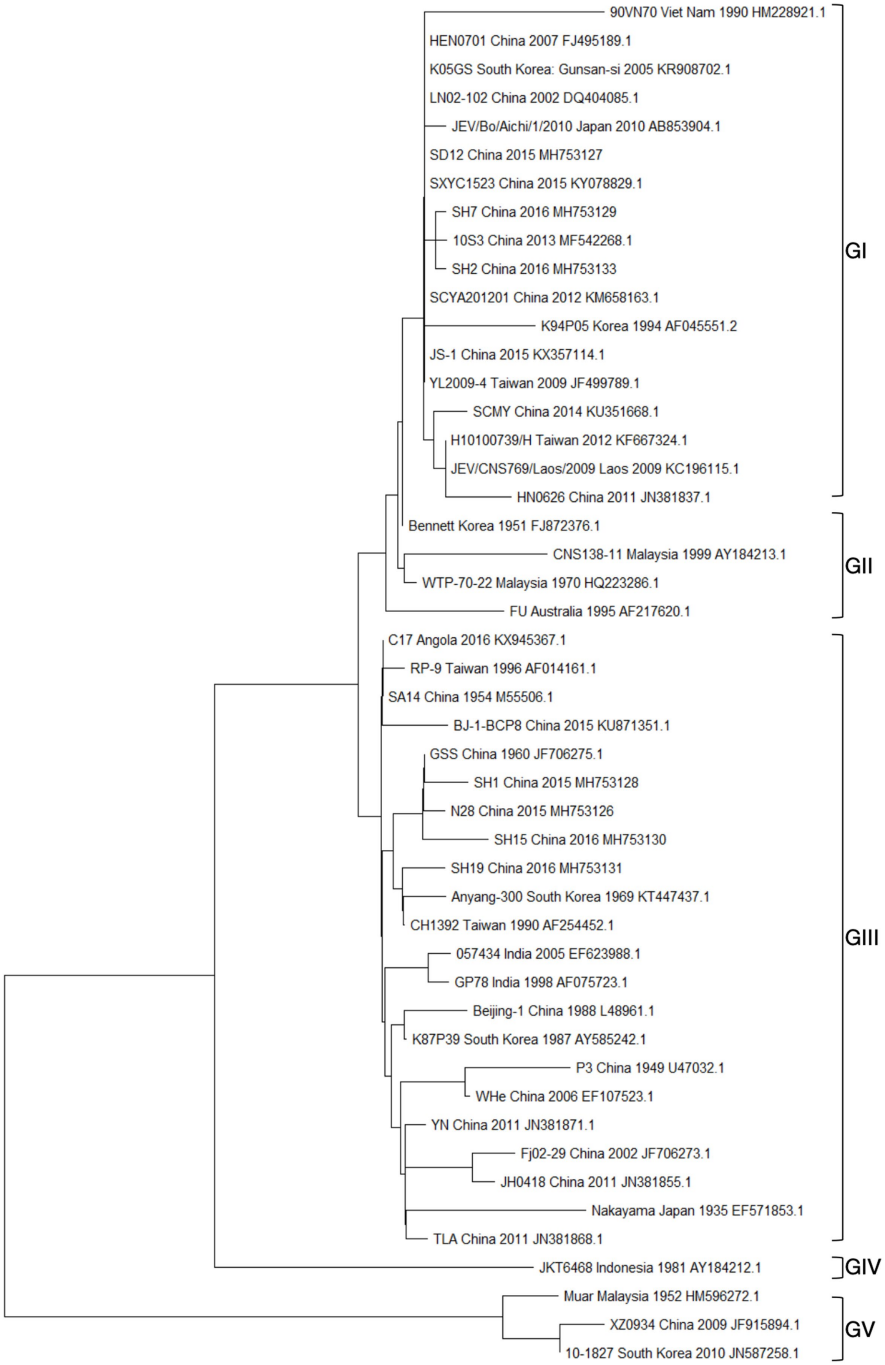
Presents the results of a phylogenetic study conducted on the JEV. This analysis was based on the complete nucleic acid sequences of E genes.

The collection of JEV sequences from GenBank, with the exclusion of duplicate and derivative sequences. Representative strains were carefully chosen, and a phylogenetic tree was then created (Figure 3; Supplementary Table S1). In most countries where JEV is endemic, more than one genotype may be transmitted simultaneously, and the predominant genotype may change periodically (Beasley et al., 2008). Currently, three genotypes of JEV have been isolated and identified in China, namely GI, GIII, and GV types. Among them, the earliest one was GIII JEV, and since then, it has shown a cross epidemic of the GI and GIII types, but the GIII strains were still predominant (Wang et al., 2007). In recent years, numerous findings have indicated a shift in the prevalence of JEV genotypes in several Asian nations, such as India, Vietnam, Malaysia, Japan, Korea, Thailand, and China. Specifically, the GI genotype has emerged as the predominant isolated genotype, supplanting the formerly prevalent GIII genotype (Pan et al., 2011; Han et al., 2014).

The ducklings who were administered GI exhibited elevated levels of viremia titers and demonstrated a comparatively extended duration in comparison to those that received the GIII vaccination. A total of 36 amino acid variations have been identified between GI and GIII viruses, potentially contributing to the heightened reproduction efficacy observed in GI viruses within waterfowl populations. Based on the aforementioned findings, it is postulated that the enhanced replication efficiency of GI viruses in waterfowl may potentially augment the susceptibility of mosquitoes to infection. Ultimately, this could lead to the displacement of GIII viruses by GI viruses, thereby establishing the latter as the prevailing genotype (Xiao et al., 2018a; Hameed et al., 2019, 2021). The formation of interferon (IFN)-α and β was found to be much lower in GI strains compared to GIII strains. This observation is consistent with the higher replication efficiency exhibited by GI strains in comparison to GIII strains. This enhanced inhibitory effect on the IFN-I-mediated antiviral response signifies the heightened antagonistic potential of GI strains. The replication and host adaptation advantages of GI strains in waterfowls surpass those of GIII strains. The aforementioned findings offer novel perspectives on the molecular underpinnings of the genotype shift observed in JEV (Li et al., 2020).

GIV JEV was initially isolated from mosquitoes in Indonesia in the 1980s (Solomon et al., 2003), but recently, GIV JEV seems to have become active again in nature. Since 2020, Indonesia has reported several GIV JEV isolates (Kuwata et al., 2020; Pyke et al., 2020; Faizah et al., 2021). Subsequently, GIV triggered a large-scale outbreak in Australia from 2021–2022 (Mackenzie et al., 2022; Sikazwe et al., 2022). According to Xu et al. (2023), the initial occurrence of human viral encephalitis attributed to GIV JEV resulted in a cumulative count of 47 cases and 7 fatalities as of November 2022. Furthermore, the genome of the GV JEV was detected in *Cx. tritaeniorhynchus* mosquitoes in China in 2009 (XZ0934) as reported by Li et al. (2011). Subsequently, it was also found in Culex orientalis and *Cx. pipiens* mosquitoes in South Korea in 2010 and 2012, respectively (Kim H. et al., 2015). The biological characteristics and pathogenicity of the GV strain exhibited notable distinctions when compared to the GI and GIII viruses (Tajima et al., 2015). These findings indicate the possibility that mosquitoes may be involved in the natural transmission cycle of GV JEV. The re-emergence of GV JEV after 50 years and whether it will spread further to traditionally JEV-endemic areas through an epidemic pathway similar to GI JEV will also require continuous monitoring. Before 2000, Korea and Japan kept the number of JE cases below 10 per year. However, Korea reported 129 JE cases from 2010 to 2015 (Sunwoo et al., 2016). Additional research is necessary to determine the potential correlation between the resurgence of JE in Korea and the presence of the GV strain of the JEV. Therefore, GV JEV may also arise in other endemic regions, and more attention and research are needed on the JEV cycle dynamics in these regions.

## 4 Cross-protection and antigen differences of the different genotypes of JEV

Over the past two decades, there has been a progressive shift from GIII JEV supremacy to GI in several Asian countries. The observed alteration in genotype prompts apprehension regarding the effectiveness of JE vaccinations, given that the presently authorized JE vaccines are sourced exclusively from GIII strains (Fan et al., 2012, 2013; Nah et al., 2015; Feroldi et al., 2017). In a study, Fan et al. (2012) examined the impact of genotype replacement on the viral neutralizing efficacy of GIII and GI viruses following vaccination. The researchers observed that the GIII JEV vaccine exhibited a considerably diminished ability to generate neutralizing antibodies against GI JEV (<1:10), in comparison to its efficacy against the GIII type (1:30–1:90). Liu et al. (2011) conducted a study to evaluate the protective efficacies of two types of JEV vaccines, namely the JE LAV SA14-14-2 virus and the IPV, which are currently employed in China for illness prevention. The researchers employed mice as experimental subjects and exposed them to different JEV isolates to assess the vaccines’ effectiveness. The results of this study indicate that both the LAV (with a viral load of ≥234 pfu) and the IPV (at a dilution of 1:5) vaccinations provided significant protection against all 16 tested isolates when administered by intraperitoneal (i.p.) challenge. Nevertheless, it was noted that when mice were vaccinated and subsequently exposed to intracerebral (i.c.) injection, a significant level of protection was achieved in over 60% of mice vaccinated with the LAV against most JEV isolates. Conversely, the IPV only protected less than 40% of vaccinated animals in the same challenge scenario.

Since the GI strains have replaced the GIII strains as the predominant virus strain, the development of vaccines based on the GI strains has become a hot topic in the field of JEV research. Korean researchers are currently engaged in the development of a novel live attenuated JE vaccine. This vaccine is derived from a virus that was isolated from the blood of an infected piglet in 1999, specifically known as the KV1899 strain. The novel vaccine elicited notably elevated levels of neutralizing antibodies in porcine subjects, and its efficacy and safety are currently under assessment across other animal species. Simultaneously, the production of GI KV1899 strain LAV in African monkey kidney cells, specifically Vero cells, was also undertaken (Nah et al., 2015). The production of a live attenuated GI strain (SD12-F120) involved the repeated passages of its virulent parental strain, SD12, in suckling mice and BHK-21 cells. The administration of this strain conferred full protection against the SD12 challenge in inoculated mice, suggesting its potential as a candidate strain for the development of an attenuated vaccine against Japanese encephalitis of genotype I (Anwar et al., 2020). Furthermore, Wei et al. (2019) conducted vaccination-challenge protection experiments to assess the cross-protective effectiveness of vaccines generated from genotypes GI or GIII against the challenge of a different genotype, utilizing a mouse challenge paradigm. The findings of this study indicate that the levels of neutralizing antibodies targeting GI viruses were comparatively lower in pigs immunized with vaccines produced from GIII viruses, in comparison to the levels observed for antibodies targeting GIII viruses. The vaccines produced from GIII were shown to be ineffective in providing the vaccinated mice with full protection against the challenge posed by heterologous genotype GI strains. Furthermore, the administration of a GI-inactivated vaccine to mice resulted in relatively limited efficacy in protecting against the exposure to heterologous genotype GIII strains. The observed phenomenon of partial cross-protection between GI and GIII viruses indicates the possibility of requiring novel techniques for JE vaccination. These strategies may include the development of a bivalent vaccine that may effectively control infections caused by both genotypes. Hence, it is imperative to develop a novel JE vaccine strategy that effectively tackles the limited cross-protective efficacy of current JE vaccines against diverse genotypes, particularly when considering the use of JEV natural hosts.

Furthermore, it is imperative to assess the cross-protective efficacy of existing vaccines against both GI and GIII JEV, considering the development of GV JEV. According to Cao et al. (2016), the current JE vaccine, which is based on the GIII genotype, does not offer sufficient protection against the newly developing GV JEV genotype and only induces modest levels of neutralizing/protective antibodies to GV JEV. Furthermore, an additional study revealed that IgG antibodies elicited against the GV JEV XZ0934 variant had little capacity to neutralize GIII JEV (de Wispelaere et al., 2015). The results of this study suggest that GV JEV may possess unique antigenic characteristics compared to other JEV genotypes. Consequently, the currently available JE vaccines generated from GIII may not offer sufficient protection against GV JEV. A recombinant chimeric JEV was created by incorporating structural proteins from a highly pathogenic GV strain into a weakened GI strain as the foundational framework. This study investigated the impact of cross-protection efficacy on various genotypes in mice. The findings revealed diminished cross-neutralizing antibody titers and decreased cross-protection between the GV genotype and other genotypes (Xia et al., 2022). In addition, due to the GIV JEV outbroke in Australia, further experiments are needed to evaluate the efficiency of current GIII JE vaccines against GIVJEV. According to Xu et al. (2023), there are 26 E protein amino acid site differences between emerging GIV JEV isolates and vaccine strain P3, so further experiments are needed to confirm whether mutations in these key sites affect the effect of GIII JEV vaccine on the protection of emerging GIV JEV.

**Figure 4:**
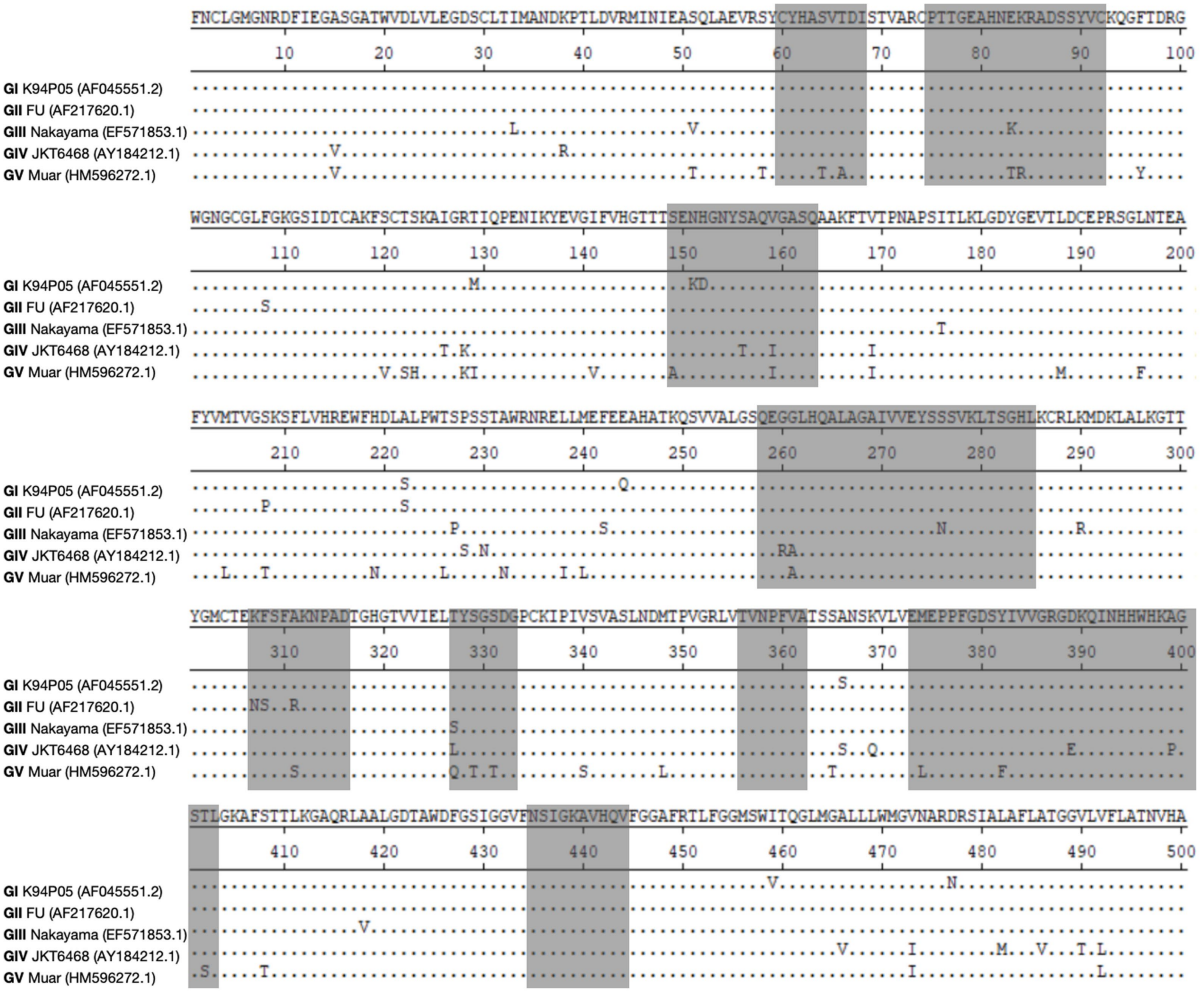
Illustrates the changes in amino acids within the E protein among different strains of genotypes. The objective of this study is to perform sequence alignment of the E proteins derived from strains GI, GII, GIII, GIV, and GV. The regions containing antigenic epitopes are visually shown by shading them in gray.

The aforementioned data indicate that the limited cross-protective and neutralizing effects observed between various genotypes may be attributed to the low homology of the E protein with other genotypes (Mohammed et al., 2011; Li et al., 2014; Tajima et al., 2015). The amino acid and nucleotide homology of the E gene among the classic strains of the five genotypes of the Japanese encephalitis virus (JEV) were studied. The analysis revealed that the amino acid homology between GI and the other four genotypes (GII, GIII, GIV, and GV) ranged from 97.6 to 90.6% (Table 1). Among them, the amino acid homology with GIII is 96.4%, with a difference of approximately 3.7% (Table 1), and the nucleotide difference is 14% (Table 2). The amino acid homology between GI and GV was 90.6%, the difference was approximately 10.1% (Table 1), and the nucleotide difference was 29.2% (Table 2). Interestingly, the amino acid difference between GV JEV and the other four genotypes was significantly higher than that between the other several genotypes; the difference was between 9.4 and 10.1% (Table 1), and the nucleotide difference was between 27 and 29.2% (Table 2). This may be the main reason why other JEV genotype-derived vaccines are less protective against GV JEV (Tajima et al., 2015). Furthermore, it was noted that certain differences in amino acids were exclusive to strains of certain genotypes. These variations were found at various locations within the E protein, encompassing epitope areas and immunodominant bait epitopes (Figure 4). These findings suggest that these variations may play a significant role in the detection of antibodies, the pathogenicity of JEV strains, and their ability to evade the immune response. The presence of distinctive amino acid changes in the E protein implies that the antigenicity of JEV strains may exhibit dissimilarities when compared to other genotypes. This phenomenon could be attributed to the fact that the effectiveness of the JEV vaccine is directly associated with the existence of neutralizing antibodies within the host. Neutralizing antibodies are mostly generated in response to the E protein, which encompasses B and T cell epitopes and impedes viral attachment to cellular receptors and fusion with the cell membrane. This may be the reason why the current JE vaccine does not provide complete protection against other genotypes of JEV.

**Table 1:** Homology of the E protein amino acids in Japanese encephalitis virus strains of different genotypes.

	K94P05 (GI)		FU (GII)		Nakayama (GIII)		JKT6468 (GIV)		Muar (GV)	
	Percent identity	Divergence	Percent identity	Divergence	Percent identity	Divergence	Percent identity	Divergence	Percent identity	Divergence
K94P05 (GI)	-	-	97.6%	2.4%	96.4%	3.7%	94.4%	5.8%	90.6%	10.1%
FU (GII)	97.6%	2.4%	-	-	96.8%	3.3%	94.4%	5.8%	91.2%	9.4%
Nakayama (GIII)	96.4%	3.7%	96.8%	3.3%	-	-	93.8%	6.5%	90.6%	10.1%
JKT6468 (GIV)	94.4%	5.8%	94.4%	5.8%	93.8%	6.5%	-	-	90.6%	10.1%
Muar (GV)	90.6%	10.1%	91.2%	9.4%	90.6%	10.1%	90.6%	10.1%	-	-

**Table 2:** Homology of the E protein nucleotides in Japanese encephalitis virus strains of different genotypes.

	K94P05 (GI)		FU (GII)		Nakayama (GIII)		JKT6468 (GIV)		Muar (GV)	
	Percent identity	Divergence	Percent identity	Divergence	Percent identity	Divergence	Percent identity	Divergence	Percent identity	Divergence
K94P05 (GI)	-	-	88.9%	12.3%	87.5%	14%	81.6%	22%	76.1%	29.2%
FU (GII)	88.9%	12.3%	-	-	88.1%	13.2%	76.1%	29.2%	77%	27.9%
Nakayama (GIII)	87.5%	14%	88.1%	13.2%	-	-	82.9%	20.2%	77.6%	27%
JKT6468 (GIV)	81.6%	22%	76.1%	29.2%	82.9%	20.2%	-	-	77.1%	27.7%
Muar (GV)	76.1%	29.2%	77%	27.9%	77.6%	27%	77.1%	27.7%	-	-

## 5 Research trends and usage of JEV vaccines

The findings from molecular epidemiological surveillance indicate a notable transition in the prevailing genotype of JEV in China, with a steady shift from GIII to GI. Additionally, it has been observed that a more virulent and pathogenic strain, GV, has evolved in the region of Tibet (Hameed et al., 2019). The prevailing genotypic alteration of JEV has resulted in an escalated risk of transmission and presents a novel obstacle for the field of public health in terms of prevention and control measures (Zheng et al., 2012; Schuh et al., 2014). Given the absence of particular pharmaceutical interventions for the treatment of JEV infection on a global scale, the implementation of immunization prophylaxis via extensive vaccination campaigns is an efficacious strategy for mitigating the transmission of JEV (Geiss et al., 2009; Sampath and Padmanabhan, 2009; Noble et al., 2010; Botting and Kuhn, 2012).

Currently, there are four different types of JE vaccines available worldwide: (1) mouse-brain-derived inactivated JE vaccines, (2) cell culture-derived live attenuated vaccines, (3) cell culture-derived inactivated vaccines, and (4) live attenuated chimeric vaccines (Beasley et al., 2008; Wilder-Smith and Halstead, 2010; Halstead and Thomas, 2011). The disadvantage of the mouse brain-purified JE vaccine is that the duration of induction of protective immunity is too short and multiple vaccinations are required (Poland et al., 1990). For most countries, the price per dose of vaccine is relatively high. Cell-cultured vaccines are produced and widely used in China, and inactivated vaccines are gradually being replaced with LAVs. Inactivated vaccines developed in mouse brain or cell culture, LAVs, and chimeric vaccines based on live attenuated yellow fever virus 17D vaccine strains are the most common types of vaccinations (Table 3). Several other promising candidates for JE are in the late stages of development. The live attenuated JE vaccine SAl4-14-2 was successfully developed in 1989 (Yu et al., 1973). In China, the adoption of the SA14-14-2 vaccine strain considerably reduced the prevalence of JE. Inoculating and passaging the SAl4-14-2 strain in suckling mice was utilized to verify its immunological stability; nucleotide sequencing and other investigations were also used. According to the findings, the SA14-14-2 strain has strong virulence stability, and its attenuation features necessitate many mutation events (Aihara et al., 1991; Ni et al., 1994, 1995; Chambers et al., 2007).

**Table 3:** Presents a comprehensive overview of four distinct categories of JE vaccinations.

The type of vaccine	JEV strain	Generic name, if any	Dose	Applicable area
Mouse brain-derived killed-inactivated	Nakayama	JE-MB	3	European Union, India, Korea, Thailand, Vietnam, Japan, Malaysia, Sri Lanka, United States
	Beijing-1	JE-MB, BK-VJE		
Cell culture-derived killed-inactivated	SA14-14-2	JE-PIV, IC51, JE-VC	2	Australia, Bangladesh, Bhutan, Canada, European Union, India, Japan, Latin America, Nepal, New Zealand, Pacific Islands, Papua New Guinea, Singapore, South Korea, Switzerland, United States
	Beijing-1	BK-VJE	3	Japan, South Korea
	Beijing-3		1/year	China
Cell culture-derived live-attenuated	SA14-14-2	SA-14–14-2	1–2	China, Cambodia, India, Laos, Myanmar, Nepal, North Korea, South Korea, Sri Lanka, etc.
Cell culture-derived live-attenuated chimeric	YFV 17D containing JEV proteins	ChimeriVax-JE; JE-CV	1–2	Australia, South Korea, Thailand

## 6 Conclusion

The GIII type of JEV predominated in prevalence from the 1930s until the end of the 20th century, but during the last 20 years, genotype displacement has occurred in JEV popularity in China and even Asia, with GI JEV gradually replacing the GIII type as the most common strain. In the 1980s, GIV JEV was first discovered from mosquitoes in Indonesia; nevertheless, in 2022, an outbreak happened in Australia (Sikazwe et al., 2022). The sequence of the emerging GIV JEV has high mutation rates, which can enhance the adaptability, competitiveness, and pathogenicity of the virus. This element perhaps plays a role in the emergence of GIV JEV infection in Australia (Xu et al., 2023). The initial variant of GV JEV, known as Muar, was initially obtained from individuals suffering from encephalitis in Malaysia in 1952 (Solomon et al., 2003). Subsequent research has revealed that this strain exhibits distinct genetic and serological characteristics compared to other genotypes. From 2009 to 2010, other GV JEVs were identified in China (Li et al., 2014) and South Korea (Takhampunya et al., 2011). The re-emergence of the GV JEV after 50 years suggests that the prevalence of the GV JEV is increasing and may also appear in other endemic areas.

Presently, the global approach to the prevention and management of JE mostly revolves around the utilization of the GIII strain vaccine for vaccination purposes. However, studies have shown that the antibodies induced by the GIII strain vaccine have a low neutralization ability to the GI strain, and cannot completely protect humans and animals from the GI strain of JEV infection. The GI strain JEV was reportedly isolated in humans immunized with the GIII strain vaccine (Zhang et al., 2011; Fan et al., 2012, 2013). In addition, the GIII strain vaccine failed to produce sufficient levels of neutralizing antibodies and protective effects against GV JEV. The displacement of the dominant strain of JEV has created a need for novel JE vaccines, and the commercial production of highly efficient GI JEV and GV JEV vaccines is expected. The displacement of the dominant genotype of the virus and the expansion of the epidemic range has brought new challenges to the prevention and control of JE. Therefore, it is necessary to accelerate the development of vaccines of different genotypes to deal with the infection caused by the JEV.

## Author contributions

QX: Writing – original draft, Writing – review & editing. YY: Writing – review & editing. YZ: Writing – review & editing. LZ: Writing – review & editing. XM: Writing – review & editing. CX: Writing – review & editing. JZ: Writing – review & editing. ZL: Writing – review & editing. KL: Writing – review & editing. BL: Writing – review & editing. DS: Writing – review & editing. YQ: Writing – review & editing. JW: Writing – review & editing. ZM: Writing – review & editing.
