# Supplementary material for: Shift in dominant genotypes of Japanese encephalitis virus and its impact on current vaccination strategies
Source: Front Microbiol. 2023 Nov 17;14:1302101. doi: 10.3389/fmicb.2023.1302101 (PMC10690641; doi:10.3389/fmicb.2023.1302101)
Supplement: Supplementary file 1 [file Table_1.DOCX]

**S1 Table 1. Information of JEV strains used for the phylogenetic tree**

| Genotype | Strains | GenBank No. | Source | Date | Country/region |
| --- | --- | --- | --- | --- | --- |
| GI | 90VN70 | HM228921.1 | *Homo sapiens* | 1990 | Viet Nam |
|  | HEN0701 | FJ495189.1 | Swine | 2007 | China |
|  | K05GS | KR908702.1 | *Culex tritaeniorhynchus* | 2005 | South Korea |
|  | LN02-102 | JF706278.1 | *Culex modestus* | 2002 | China |
|  | JEV/Bo/Aichi/1/2010 | AB853904.1 | *Bos taurus* | 2010 | Japan |
|  | SD12 | MH753127 | Pig | 2015 | China |
|  | SXYC1523 | KY078829.1 | *Culex pipiens pallens* | 2015 | China |
|  | SH7 | MH753129 | *Culex tritaeniorhynchus* | 2016 | China |
|  | 10S3 | MF542268.1 | Pig | 2013 | China |
|  | SH2 | MH753133 | *Culex tritaeniorhynchus* | 2016 | China |
|  | SCYA201201 | KM658163.1 | Pig | 2012 | China |
|  | K94P05 | AF045551.2 | *Culex tritaeniorhynchus* | 1994 | Korea |
|  | JS-1 | KX357114.1 | *Culex tritaeniorhynchus* | 2015 | China |
|  | YL2009-4 | JF499789.1 | *mosquito* | 2009 | Taiwan |
|  | SCMY | KU351668.1 | Pig | 2014 | China |
|  | H10100739/H | KF667324.1 | *Homo sapiens* | 2012 | Taiwan |
|  | JEV/CNS769/Laos/2009 | KC196115.1 | *Homo sapiens* | 2009 | Laos |
|  | HN0626 | JN381837.1 | *Culex* | 2011 | China |
| GII | Bennett | FJ872376.1 | *Homo sapiens* | 1951 | Korea |
|  | CNS138-11 | AY184213.1 | Human brain | 1999 | Malaysia |
|  | WTP-70-22 | HQ223286.1 | Mosquito | 1970 | Malaysia |
|  | FU | AF217620.1 | *Homo sapiens* | 1995 | Australia |
| GIII | C17 | KX945367.1 | *Homo sapiens* | 2016 | Angola |
|  | RP-9 | AF014161.1 | *Culex tritaeniorhynchu*s | 1996 | Taiwan |
|  | SA14 | M55506.1 | Mosquito | 1954 | China |
|  | BJ-1-BCP8 | KU871351.1 | Unknown | 2015 | China |
|  | GSS | JF706275.1 | Pig | 1960 | China |
|  | SH1 | MH753128 | Pig | 2015 | China |
|  | N28 | MH753126 | Pig | 2015 | China |
|  | SH15 | MH753130 | *Anopheles sinensis* | 2016 | China |
|  | SH19 | MH753131 | *Anopheles sinensis* | 2016 | China |
|  | Anyang-300 | KT447437.1 | Pig | 1969 | South Korea |
|  | CH1392 | AF254452.1 | *Culex tritaeniorhynchus* | 1990 | Taiwan |
|  | 057434 | EF623988.1 | *Homo sapiens* | 2005 | India |
|  | GP78 | AF075723.1 | *Homo sapiens* | 1998 | India |
|  | Beijing-1 | L48961.1 | *Homo sapiens* | 1988 | China |
|  | K87P39 | AY585242.1 | Mosquito | 1987 | South Korea |
|  | P3 | U47032.1 | *Homo sapiens* | 1949 | China |
|  | WHe | EF107523.1 | Pig | 2006 | China |
|  | YN | JN381871.1 | *Homo sapiens* | 2011 | China |
|  | Fj02-29 | JF706273.1 | Pig | 2002 | China |
|  | JH0418 | JN381855.1 | *Culex whitmorei and Anopheles sinensis* | 2011 | China |
|  | Nakayama | EF571853.1 | *Homo sapiens* | 1935 | Japan |
|  | TLA | JN381868.1 | *Homo sapiens* | 2011 | China |
| GⅣ | JKT6468 | AY184212.1 | mosquito | 1981 | Indonesia |
| GⅤ | Muar | HM596272.1 | *Homo sapiens* | 1952 | Malaysia |
|  | XZ0934 | JF915894.1 | Mosquito | 2009 | China |
|  | 10-1827 | JN587258.1 | *Culex tritaeniorhynchus* | 2010 | South Korea |
